# 7-Methyl-1-phenyl-1,10-di­hydro­pyrazolo­[3,4-*a*]carbazole

**DOI:** 10.1107/S1600536813010994

**Published:** 2013-04-27

**Authors:** R. Archana, E. Yamuna, A. Thiruvalluvar, K. J. Rajendra Prasad, R. J. Butcher, Sushil K. Gupta, Sema Öztürk Yildirim

**Affiliations:** a Postgraduate Research Department of Physics, Rajah Serfoji Government College (Autonomous), Thanjavur 613 005, Tamilnadu, India; b Department of Chemistry, Tamkang University, Tamsui 25137, Taiwan; c Department of Chemistry, Bharathiar University, Coimbatore 641 046, Tamilnadu, India; d Department of Chemistry, Howard University, 525 College Street NW, Washington, DC 20059, USA; e School of Studies in Chemistry, Jiwaji University, Gwalior 474 011, MP, India; f Faculty of Sciences, Department of Physics, Erciyes University, 38039 Kayseri, Turkey

## Abstract

In the title mol­ecule, C_20_H_15_N_3_, the atoms in the carbazole unit deviate from planarity [maximum deviation from mean plane = 0.1082 (15) Å]. The pyrrole ring makes dihedral angles of 3.17 (8)/4.10 (9), 7.20 (9) and 44.62 (9)° with the fused benzene, pyrazole and phenyl rings, respectively. In the crystal, mol­ecules are linked *via* N—H⋯N hydrogen bonds, forming an infinite chain along [010]. Mol­ecules are further linked by nine π–π [centroid–centroid distances vary from 3.6864 (11) to 3.9802 (11) Å] and one C—H⋯π inter­action, forming a three-dimensional network.

## Related literature

For related structures and the biological and pharmacological activity of carbazole alkaloids, see: Archana *et al.* (2010 ▶, 2011 ▶).
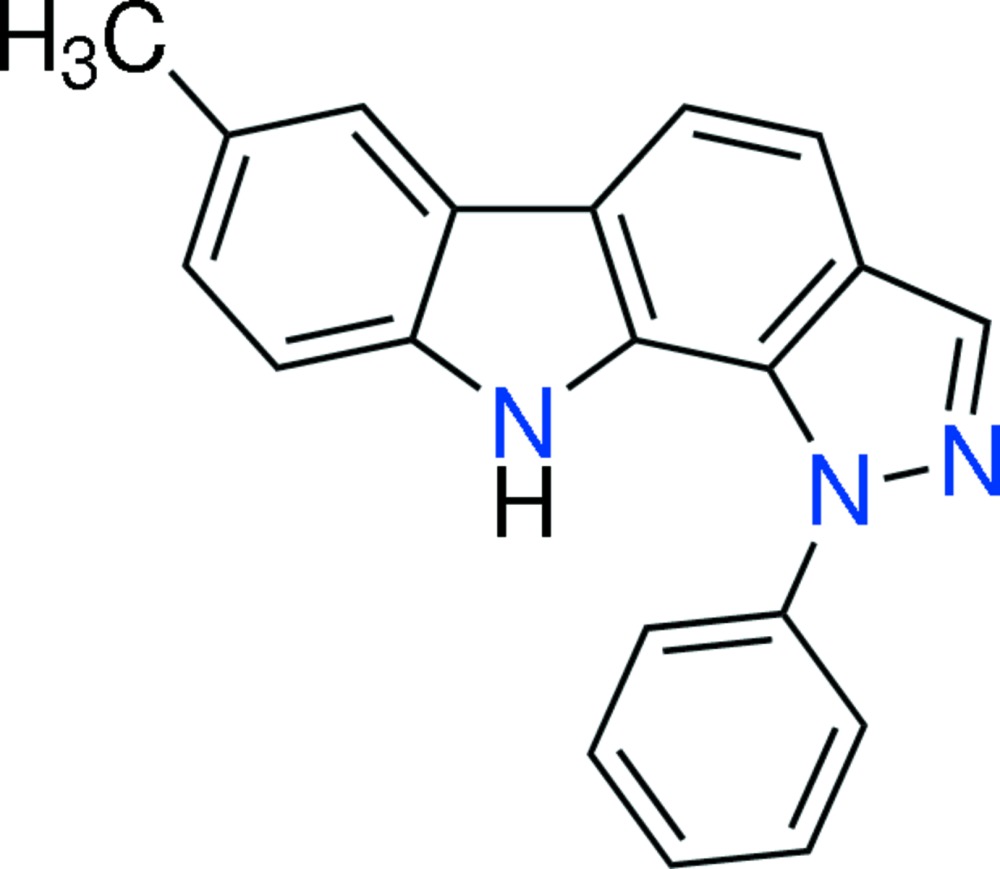


## Experimental

### 

#### Crystal data

C_20_H_15_N_3_
*M* *_r_* = 297.35
Monoclinic, 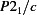

*a* = 12.0727 (6) Å
*b* = 7.5934 (3) Å
*c* = 16.8355 (8) Å
β = 104.087 (5)°
*V* = 1496.95 (12) Å^3^
*Z* = 4
Mo *K*α radiation
μ = 0.08 mm^−1^
*T* = 123 K
0.43 × 0.35 × 0.30 mm

#### Data collection

Agilent Xcalibur Ruby Gemini diffractometer
Absorption correction: multi-scan (*CrysAlis PRO*; Agilent, 2011 ▶) *T*_min_ = 0.967, *T*_max_ = 0.977
6773 measured reflections
3212 independent reflections
2354 reflections with *I* > 2σ(*I*)
*R*_int_ = 0.032

#### Refinement

*R*[*F* ^2^ > 2σ(*F* ^2^)] = 0.051
*wR*(*F* ^2^) = 0.115
*S* = 1.03
3212 reflections
213 parameters
H atoms treated by a mixture of independent and constrained refinement
Δρ_max_ = 0.24 e Å^−3^
Δρ_min_ = −0.25 e Å^−3^

### 

Data collection: *CrysAlis PRO* (Agilent, 2011 ▶); cell refinement: *CrysAlis PRO*; data reduction: *CrysAlis PRO*; program(s) used to solve structure: *SHELXS97* (Sheldrick, 2008 ▶); program(s) used to refine structure: *SHELXL97* (Sheldrick, 2008 ▶); molecular graphics: *ORTEP-3 for Windows* (Farrugia, 2012 ▶) and *PLATON* (Spek, 2009 ▶); software used to prepare material for publication: *SHELXL97* and *PLATON*.

## Supplementary Material

Crystal structure: contains datablock(s) global, I. DOI: 10.1107/S1600536813010994/bg2506sup1.cif

Structure factors: contains datablock(s) I. DOI: 10.1107/S1600536813010994/bg2506Isup2.hkl

Supplementary material file. DOI: 10.1107/S1600536813010994/bg2506Isup3.cdx

Supplementary material file. DOI: 10.1107/S1600536813010994/bg2506Isup4.cml

Additional supplementary materials:  crystallographic information; 3D view; checkCIF report

## Figures and Tables

**Table 1:** Hydrogen-bond geometry (Å, °) *Cg*2 is the centroid of the N10/C10*A*/C5*A*/C5*B*/C9*A* pyrrole ring.

*D*—H⋯*A*	*D*—H	H⋯*A*	*D*⋯*A*	*D*—H⋯*A*
N10—H10⋯N2^i^	0.89 (2)	2.24 (2)	3.092 (2)	159 (2)
C17—H17*B*⋯*Cg*2^ii^	0.98	2.70	3.401 (2)	129

Symmetry codes: (i) 
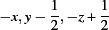
; (ii) 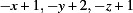.

**Fig. 1.**
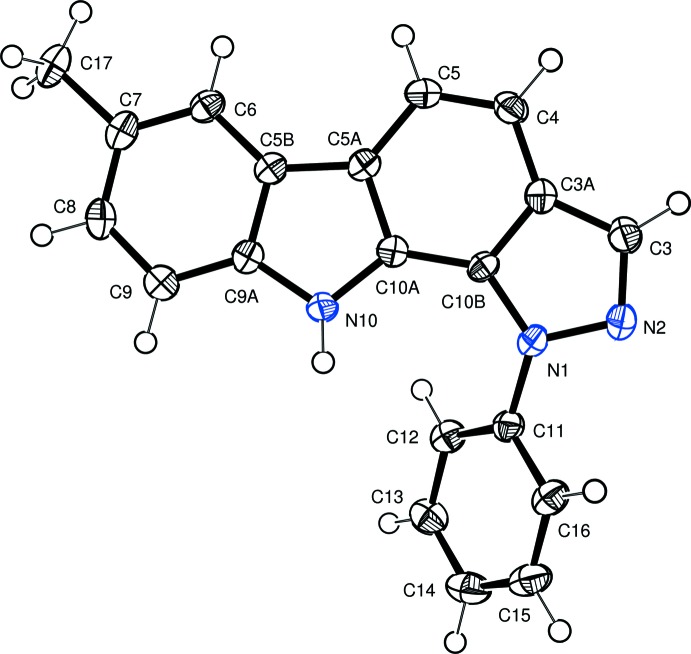
The molecular structure of the title compound, with displacement ellipsoids drawn at the 50% probability level. H atoms are shown as small spheres of arbitrary radius.

**Fig. 2.**
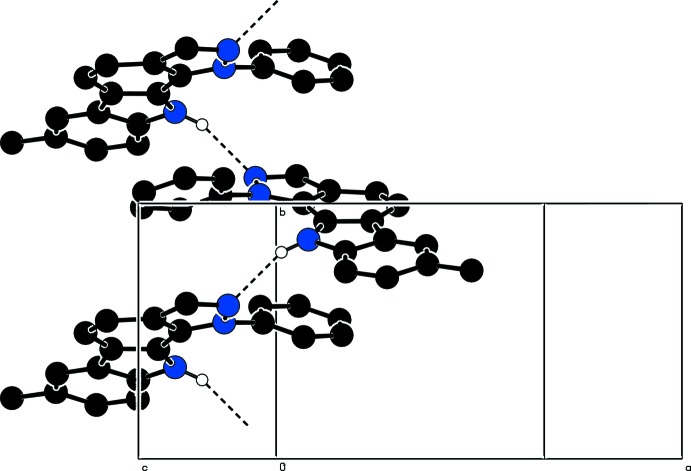
The partial packing of the title compound, viewed approximately down the c axis. Dashed lines indicate hydrogen bonds. H atoms not involved in hydrogen bonding have been omitted.

**Fig. 3.**
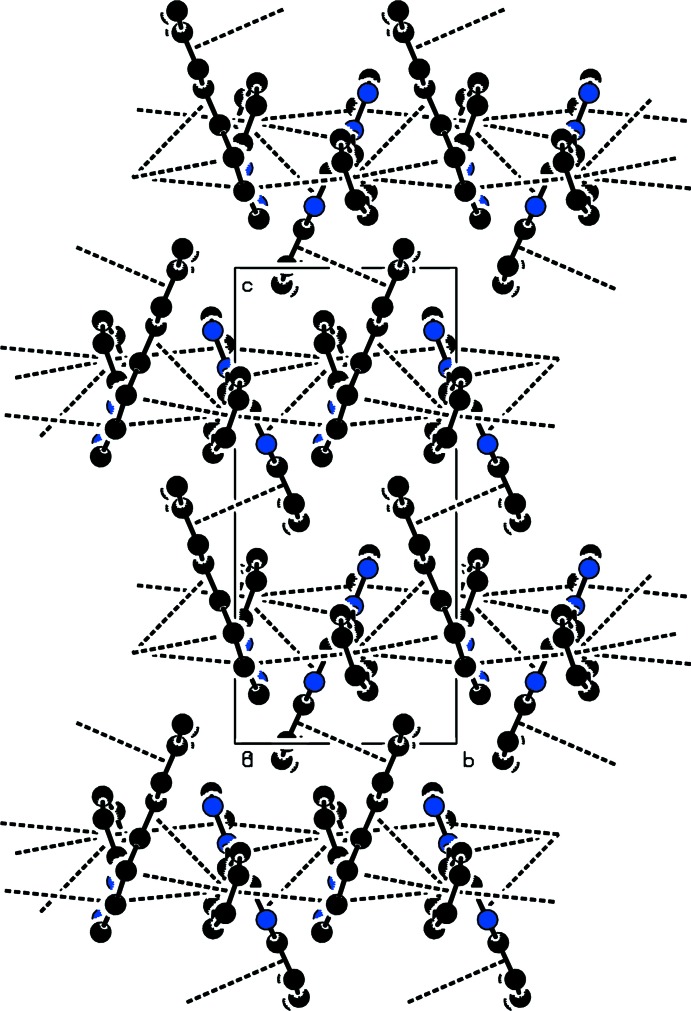
The crystal structure of compound, showing the formation of π-π stacking interactions.

**Fig. 4.**
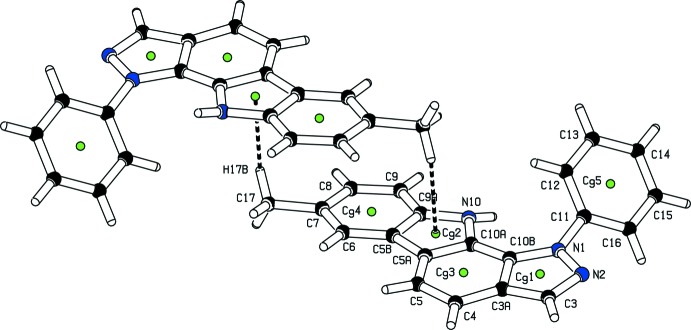
Part of the crystal structure of compound, showing the formation of C—H···π interactions. Symmetry code ii: 1 - x, 2 - y, 1 - z

## supplementary crystallographic information

### Comment

As part of our research (Archana *et al.*, 2010, 2011), we have synthesized the title compound (I), and report its crystal structure here.

In the title molecule (Scheme I, Fig. 1), C_20_H_15_N_3_, the atoms in the carbazole unit deviate from planarity. Maximum deviation from carbazole mean plane = -0.1082 (15) Å for atom C4. The pyrrole ring makes dihedral angles of 3.17 (8), 4.10 (9), 7.20 (9) and 44.62 (9)° with the fused benzene rings, pyrazole and phenyl rings, respectively.

In the crystal structure, molecules are linked *via* a N10—H10···N2 interaction, forming an infinite one-dimensional chain with base vector [0 1 0] (Table 1, Fig. 2). Molecules are further linked by nine π-π [*Cg*1—*Cg*5^i^ = *Cg*5—*Cg*1^iii^ = 3.9802 (11), *Cg*1—*Cg*5^iii^ = *Cg*5—*Cg*1^i^ = 3.6864 (11), *Cg*2—*Cg*5^i^ = *Cg*5—*Cg*2^iii^ = 3.9402 (11), *Cg*3—*Cg*5^i^ = 3.7920 (11), *Cg*4—*Cg*4^ii^ = 3.8456 (9) and *Cg*5—*Cg*3^iii^ = 3.7921 (11) Å, symmetry code (i): -*x*, -1/2 + *y*, 1/2 - *z*, (ii): 1 - *x*, 2 - *y*, 1 - *z*, (iii): -*x*, 1/2 + *y*, 1/2 - *z* where *Cg*1, *Cg*2, *Cg*3, *Cg*4 and *Cg*5 are the centroids of the pyrazole (N1/N2/C3/C3A/C10B), pyrrole (N10/C10A/C5A/C5B/C9A), benzene (C3A/C4/C5/C5A/C10A/C10B), benzene (C5B/C6—C9/C9A) and phenyl (C11—C16) rings, respectively (Fig. 3)] and one C17—H17B···π interactions to form a three-dimensional network (Table 1, Fig. 4).

### Experimental

A mixture of 2-(hydroxymethylene)-6-methyl-2,3,4,9-tetrahydro-1*H*- carbazol-1-one (0.227 g, 0.001 mol), phenyl hydrazine (0.540 g, 0.005 mol) and glacial acetic acid (5 ml) was refluxed at 393 K for 6 h. After completion of reaction it was then cooled and poured onto crushed ice, the solid thus separated out was filtered, washed with water, dried and purified by column chromatography over silica gel (eluting with a petroleum ether and ethyl acetate mixture, 95:5) to give the title compound (0.228 g, 77%). This pure compound was recrystallized from EtOAc and ethanol.

### Refinement

The H atom bonded to N10 was located in a difference Fourier map and refined freely; N10—H10 = 0.89 (2) Å. Other H atoms were positioned geometrically and allowed to ride on their parent atoms, with C—H = 0.95–0.98 Å, and with *U*_iso_(H) = 1.2–1.5*U*_eq_(parent atom).

### Crystal data

**Table:**

C_20_H_15_N_3_	*F*(000) = 624
*M**_r_* = 297.35	*D*_x_ = 1.319 Mg m^−^^3^
Monoclinic, *P*2_1_/*c*	Melting point: 509 K
Hall symbol: -P 2ybc	Mo *K*α radiation, λ = 0.71073 Å
*a* = 12.0727 (6) Å	Cell parameters from 2152 reflections
*b* = 7.5934 (3) Å	θ = 3.2–28.7°
*c* = 16.8355 (8) Å	µ = 0.08 mm^−^^1^
β = 104.087 (5)°	*T* = 123 K
*V* = 1496.95 (12) Å^3^	Block, colourless
*Z* = 4	0.43 × 0.35 × 0.30 mm

### Data collection

**Table:**

Agilent Xcalibur Ruby Gemini diffractometer	3212 independent reflections
Radiation source: Enhance (Mo) X-ray Source	2354 reflections with *I* > 2σ(*I*)
Graphite monochromator	*R*_int_ = 0.032
Detector resolution: 10.5081 pixels mm^-1^	θ_max_ = 28.8°, θ_min_ = 3.2°
ω scans	*h* = −11→15
Absorption correction: multi-scan (*CrysAlis PRO*; Agilent, 2011)	*k* = −9→9
*T*_min_ = 0.967, *T*_max_ = 0.977	*l* = −22→17
6773 measured reflections	

### Refinement

**Table:**

Refinement on *F*^2^	Primary atom site location: structure-invariant direct methods
Least-squares matrix: full	Secondary atom site location: difference Fourier map
*R*[*F*^2^ > 2σ(*F*^2^)] = 0.051	Hydrogen site location: inferred from neighbouring sites
*wR*(*F*^2^) = 0.115	H atoms treated by a mixture of independent and constrained refinement
*S* = 1.03	*w* = 1/[σ^2^(*F*_o_^2^) + (0.0374*P*)^2^ + 0.6346*P*] where *P* = (*F*_o_^2^ + 2*F*_c_^2^)/3
3212 reflections	(Δ/σ)_max_ = 0.001
213 parameters	Δρ_max_ = 0.24 e Å^−^^3^
0 restraints	Δρ_min_ = −0.25 e Å^−^^3^

### Special details

**Table:**

Geometry. Bond distances, angles *etc*. have been calculated using the rounded fractional coordinates. All su's are estimated from the variances of the (full) variance-covariance matrix. The cell e.s.d.'s are taken into account in the estimation of distances, angles and torsion angles
Refinement. Refinement of *F*^2^ against ALL reflections. The weighted *R*-factor *wR* and goodness of fit *S* are based on *F*^2^, conventional *R*-factors *R* are based on *F*, with *F* set to zero for negative *F*^2^. The threshold expression of *F*^2^ > 2σ(*F*^2^) is used only for calculating *R*-factors(gt) *etc*. and is not relevant to the choice of reflections for refinement. *R*-factors based on *F*^2^ are statistically about twice as large as those based on *F*, and *R*- factors based on ALL data will be even larger.

### Fractional atomic coordinates and isotropic or equivalent isotropic displacement parameters (Å^2^)

**Table:**

	*x*	*y*	*z*	*U*_iso_*/*U*_eq_	
N1	0.02985 (12)	1.03390 (19)	0.21043 (9)	0.0239 (4)	
N2	−0.00637 (13)	1.1004 (2)	0.13198 (9)	0.0282 (5)	
N10	0.20532 (12)	0.85847 (19)	0.37045 (9)	0.0218 (5)	
C3	0.08613 (15)	1.1075 (2)	0.10345 (11)	0.0279 (6)	
C3A	0.18491 (15)	1.0494 (2)	0.16110 (10)	0.0226 (5)	
C4	0.30269 (15)	1.0405 (3)	0.16171 (11)	0.0276 (6)	
C5	0.37898 (16)	0.9927 (3)	0.23251 (11)	0.0296 (6)	
C5A	0.33861 (14)	0.9308 (2)	0.30042 (11)	0.0229 (5)	
C5B	0.39645 (14)	0.8572 (2)	0.37826 (10)	0.0216 (5)	
C6	0.51173 (15)	0.8333 (2)	0.41785 (11)	0.0244 (5)	
C7	0.54120 (15)	0.7615 (2)	0.49534 (11)	0.0255 (5)	
C8	0.45442 (16)	0.7109 (2)	0.53306 (11)	0.0270 (6)	
C9	0.33971 (15)	0.7336 (2)	0.49600 (11)	0.0255 (6)	
C9A	0.31196 (14)	0.8101 (2)	0.41843 (10)	0.0217 (5)	
C10A	0.22194 (14)	0.9297 (2)	0.29845 (10)	0.0210 (5)	
C10B	0.14522 (14)	1.0011 (2)	0.22935 (10)	0.0199 (5)	
C11	−0.04799 (14)	1.0332 (2)	0.26172 (11)	0.0229 (5)	
C12	−0.01450 (16)	1.0956 (2)	0.34141 (11)	0.0263 (5)	
C13	−0.09314 (17)	1.0990 (3)	0.38894 (12)	0.0322 (6)	
C14	−0.20453 (17)	1.0443 (3)	0.35701 (13)	0.0360 (7)	
C15	−0.23685 (17)	0.9846 (3)	0.27740 (13)	0.0350 (7)	
C16	−0.15914 (15)	0.9769 (2)	0.22938 (12)	0.0290 (6)	
C17	0.66497 (15)	0.7377 (3)	0.54049 (12)	0.0315 (6)	
H3	0.08626	1.14732	0.04998	0.0334*	
H4	0.32788	1.06729	0.11384	0.0331*	
H5	0.45869	1.00054	0.23653	0.0355*	
H6	0.56955	0.86651	0.39130	0.0292*	
H8	0.47522	0.65913	0.58598	0.0324*	
H9	0.28215	0.69847	0.52242	0.0306*	
H10	0.1399 (17)	0.809 (3)	0.3743 (12)	0.034 (6)*	
H12	0.06146	1.13546	0.36304	0.0316*	
H13	−0.07056	1.13924	0.44395	0.0387*	
H14	−0.25836	1.04795	0.38980	0.0431*	
H15	−0.31347	0.94826	0.25532	0.0420*	
H16	−0.18151	0.93355	0.17487	0.0348*	
H17A	0.68418	0.61209	0.54327	0.0473*	
H17B	0.67687	0.78502	0.59609	0.0473*	
H17C	0.71401	0.80058	0.51136	0.0473*	

### Atomic displacement parameters (Å^2^)

**Table:**

	*U*^11^	*U*^22^	*U*^33^	*U*^12^	*U*^13^	*U*^23^
N1	0.0196 (7)	0.0276 (8)	0.0234 (8)	0.0030 (7)	0.0032 (6)	0.0019 (6)
N2	0.0261 (8)	0.0322 (9)	0.0243 (8)	0.0044 (7)	0.0025 (7)	0.0044 (7)
N10	0.0192 (8)	0.0238 (8)	0.0222 (8)	−0.0024 (7)	0.0046 (7)	0.0009 (6)
C3	0.0307 (10)	0.0284 (10)	0.0246 (10)	0.0024 (9)	0.0068 (8)	0.0013 (8)
C3A	0.0254 (9)	0.0205 (9)	0.0212 (9)	0.0002 (8)	0.0042 (8)	−0.0021 (7)
C4	0.0277 (10)	0.0350 (11)	0.0216 (9)	−0.0024 (9)	0.0092 (8)	0.0000 (8)
C5	0.0225 (9)	0.0362 (11)	0.0324 (11)	0.0012 (9)	0.0113 (9)	0.0046 (9)
C5A	0.0204 (9)	0.0216 (9)	0.0260 (10)	0.0005 (8)	0.0046 (8)	−0.0014 (7)
C5B	0.0217 (9)	0.0186 (8)	0.0241 (9)	0.0005 (8)	0.0050 (8)	−0.0030 (7)
C6	0.0210 (9)	0.0213 (9)	0.0302 (10)	0.0004 (8)	0.0051 (8)	−0.0028 (8)
C7	0.0269 (9)	0.0175 (9)	0.0282 (10)	0.0025 (8)	−0.0007 (8)	−0.0056 (7)
C8	0.0334 (10)	0.0212 (9)	0.0230 (10)	0.0021 (8)	0.0005 (8)	0.0000 (8)
C9	0.0288 (10)	0.0223 (9)	0.0255 (10)	−0.0016 (8)	0.0069 (8)	−0.0028 (8)
C9A	0.0226 (9)	0.0187 (9)	0.0224 (9)	−0.0008 (8)	0.0029 (8)	−0.0033 (7)
C10A	0.0220 (9)	0.0199 (9)	0.0211 (9)	−0.0006 (7)	0.0052 (8)	−0.0017 (7)
C10B	0.0170 (9)	0.0196 (9)	0.0223 (9)	0.0000 (7)	0.0031 (7)	−0.0032 (7)
C11	0.0215 (9)	0.0197 (9)	0.0279 (10)	0.0037 (8)	0.0069 (8)	0.0032 (8)
C12	0.0244 (9)	0.0232 (9)	0.0311 (10)	0.0003 (8)	0.0062 (8)	−0.0014 (8)
C13	0.0381 (11)	0.0279 (10)	0.0342 (11)	0.0022 (9)	0.0155 (9)	−0.0014 (9)
C14	0.0351 (11)	0.0308 (11)	0.0488 (13)	0.0008 (9)	0.0234 (10)	0.0013 (10)
C15	0.0230 (10)	0.0297 (10)	0.0532 (14)	−0.0016 (9)	0.0109 (10)	0.0030 (10)
C16	0.0233 (10)	0.0268 (10)	0.0348 (11)	−0.0008 (8)	0.0030 (8)	−0.0007 (8)
C17	0.0282 (10)	0.0279 (10)	0.0324 (11)	0.0064 (9)	−0.0044 (9)	−0.0059 (8)

### Geometric parameters (Å, º)

**Table:**

N1—N2	1.382 (2)	C10A—C10B	1.407 (2)
N1—C10B	1.374 (2)	C11—C16	1.387 (3)
N1—C11	1.423 (2)	C11—C12	1.387 (2)
N2—C3	1.320 (2)	C12—C13	1.383 (3)
N10—C9A	1.392 (2)	C13—C14	1.385 (3)
N10—C10A	1.386 (2)	C14—C15	1.378 (3)
N10—H10	0.89 (2)	C15—C16	1.381 (3)
C3—C3A	1.413 (2)	C3—H3	0.9500
C3A—C4	1.421 (3)	C4—H4	0.9500
C3A—C10B	1.397 (2)	C5—H5	0.9500
C4—C5	1.366 (3)	C6—H6	0.9500
C5—C5A	1.427 (3)	C8—H8	0.9500
C5A—C5B	1.439 (2)	C9—H9	0.9500
C5A—C10A	1.401 (2)	C12—H12	0.9500
C5B—C9A	1.401 (2)	C13—H13	0.9500
C5B—C6	1.401 (3)	C14—H14	0.9500
C6—C7	1.378 (2)	C15—H15	0.9500
C7—C8	1.404 (3)	C16—H16	0.9500
C7—C17	1.513 (3)	C17—H17A	0.9800
C8—C9	1.384 (3)	C17—H17B	0.9800
C9—C9A	1.394 (2)	C17—H17C	0.9800
			
N2—N1—C10B	110.79 (14)	C12—C11—C16	120.75 (17)
N2—N1—C11	118.65 (14)	N1—C11—C16	118.80 (16)
C10B—N1—C11	129.85 (15)	C11—C12—C13	119.11 (18)
N1—N2—C3	105.30 (15)	C12—C13—C14	120.56 (18)
C9A—N10—C10A	107.47 (14)	C13—C14—C15	119.63 (19)
C9A—N10—H10	123.9 (14)	C14—C15—C16	120.76 (19)
C10A—N10—H10	123.2 (13)	C11—C16—C15	119.17 (18)
N2—C3—C3A	112.63 (16)	N2—C3—H3	124.00
C4—C3A—C10B	121.63 (16)	C3A—C3—H3	124.00
C3—C3A—C4	134.09 (16)	C3A—C4—H4	121.00
C3—C3A—C10B	104.23 (16)	C5—C4—H4	121.00
C3A—C4—C5	118.68 (17)	C4—C5—H5	120.00
C4—C5—C5A	119.82 (18)	C5A—C5—H5	120.00
C5B—C5A—C10A	106.35 (15)	C5B—C6—H6	120.00
C5—C5A—C10A	121.36 (16)	C7—C6—H6	120.00
C5—C5A—C5B	132.27 (17)	C7—C8—H8	119.00
C5A—C5B—C6	133.56 (16)	C9—C8—H8	119.00
C5A—C5B—C9A	106.85 (15)	C8—C9—H9	121.00
C6—C5B—C9A	119.55 (15)	C9A—C9—H9	121.00
C5B—C6—C7	119.95 (17)	C11—C12—H12	120.00
C8—C7—C17	119.62 (16)	C13—C12—H12	120.00
C6—C7—C8	119.15 (17)	C12—C13—H13	120.00
C6—C7—C17	121.23 (17)	C14—C13—H13	120.00
C7—C8—C9	122.46 (16)	C13—C14—H14	120.00
C8—C9—C9A	117.40 (16)	C15—C14—H14	120.00
N10—C9A—C9	129.13 (16)	C14—C15—H15	120.00
N10—C9A—C5B	109.38 (14)	C16—C15—H15	120.00
C5B—C9A—C9	121.45 (16)	C11—C16—H16	120.00
C5A—C10A—C10B	118.22 (15)	C15—C16—H16	120.00
N10—C10A—C10B	131.84 (16)	C7—C17—H17A	109.00
N10—C10A—C5A	109.90 (15)	C7—C17—H17B	109.00
N1—C10B—C3A	107.04 (14)	C7—C17—H17C	109.00
N1—C10B—C10A	133.44 (16)	H17A—C17—H17B	109.00
C3A—C10B—C10A	119.50 (16)	H17A—C17—H17C	109.00
N1—C11—C12	120.38 (16)	H17B—C17—H17C	109.00
			
C10B—N1—N2—C3	0.15 (18)	C5—C5A—C10A—N10	178.07 (16)
C11—N1—N2—C3	171.43 (14)	C5—C5A—C10A—C10B	−3.7 (2)
N2—N1—C10B—C3A	0.50 (18)	C5B—C5A—C10A—N10	−0.41 (18)
N2—N1—C10B—C10A	−177.71 (17)	C5B—C5A—C10A—C10B	177.85 (14)
C11—N1—C10B—C3A	−169.53 (15)	C5A—C5B—C6—C7	178.06 (17)
C11—N1—C10B—C10A	12.3 (3)	C9A—C5B—C6—C7	0.9 (2)
N2—N1—C11—C12	−132.06 (16)	C5A—C5B—C9A—N10	−2.61 (18)
N2—N1—C11—C16	44.9 (2)	C5A—C5B—C9A—C9	179.74 (14)
C10B—N1—C11—C12	37.3 (2)	C6—C5B—C9A—N10	175.22 (14)
C10B—N1—C11—C16	−145.69 (17)	C6—C5B—C9A—C9	−2.4 (2)
N1—N2—C3—C3A	−0.76 (18)	C5B—C6—C7—C8	0.9 (2)
C10A—N10—C9A—C5B	2.37 (18)	C5B—C6—C7—C17	−178.63 (16)
C10A—N10—C9A—C9	179.78 (16)	C6—C7—C8—C9	−1.3 (2)
C9A—N10—C10A—C5A	−1.18 (18)	C17—C7—C8—C9	178.20 (16)
C9A—N10—C10A—C10B	−179.12 (16)	C7—C8—C9—C9A	−0.1 (2)
N2—C3—C3A—C4	−176.18 (19)	C8—C9—C9A—N10	−175.15 (16)
N2—C3—C3A—C10B	1.05 (18)	C8—C9—C9A—C5B	2.0 (2)
C3—C3A—C4—C5	173.19 (19)	N10—C10A—C10B—N1	4.0 (3)
C10B—C3A—C4—C5	−3.7 (3)	N10—C10A—C10B—C3A	−174.02 (16)
C3—C3A—C10B—N1	−0.89 (17)	C5A—C10A—C10B—N1	−173.79 (17)
C3—C3A—C10B—C10A	177.62 (14)	C5A—C10A—C10B—C3A	8.2 (2)
C4—C3A—C10B—N1	176.78 (16)	N1—C11—C12—C13	177.78 (16)
C4—C3A—C10B—C10A	−4.7 (2)	C16—C11—C12—C13	0.8 (2)
C3A—C4—C5—C5A	8.2 (3)	N1—C11—C16—C15	−176.60 (16)
C4—C5—C5A—C5B	173.34 (19)	C12—C11—C16—C15	0.4 (2)
C4—C5—C5A—C10A	−4.7 (3)	C11—C12—C13—C14	−1.3 (3)
C5—C5A—C5B—C6	6.2 (3)	C12—C13—C14—C15	0.6 (3)
C5—C5A—C5B—C9A	−176.42 (19)	C13—C14—C15—C16	0.7 (3)
C10A—C5A—C5B—C6	−175.56 (17)	C14—C15—C16—C11	−1.2 (3)
C10A—C5A—C5B—C9A	1.83 (17)		

### Hydrogen-bond geometry (Å, º)

Cg2 is the centroid of the N10/C10A/C5A/C5B/C9A pyrrole ring.

**Table:**

*D*—H···*A*	*D*—H	H···*A*	*D*···*A*	*D*—H···*A*
N10—H10···N2^i^	0.89 (2)	2.24 (2)	3.092 (2)	159 (2)
C17—H17*B*···*Cg*2^ii^	0.98	2.70	3.401 (2)	129

Symmetry codes: (i) −*x*, *y*−1/2, −*z*+1/2; (ii) −*x*+1, −*y*+2, −*z*+1.
